# Age Differences in Hazard Perception of Drivers: The Roles of Emotion

**DOI:** 10.3389/fpsyg.2022.867673

**Published:** 2022-06-02

**Authors:** Faren Huo, Ranran Gao, Cong Sun, Guanhua Hou

**Affiliations:** Pan Tianshou College of Architecture, Arts and Design, Ningbo University, Ningbo, China

**Keywords:** emotion, age, hazard perception, traffic warning signs, drivers

## Abstract

With the increasingly powerful functions of vehicle-mounted entertainment facilities, people (especially young drivers) like to listen to music while driving to render different atmospheres and emotions. However, emotions are important factors affecting drivers’ decisions, behavior and may reduce drivers’ hazard perception (HP), even promote dangerous driving behaviors of drivers. The purpose of this study is to explore the young and elderly drivers in assessing the HP difference under different emotional states. We conducted a 3 × 2 mixed experimental design with emotion as a within-participants variable and age as a between-participants factor. A sample of 14 young drivers (mean age = 22.21, SD = 1.05) and 13 elderly drivers (mean age = 54.08, SD = 2.72) completed the HP self-assessment of road traffic warning signs under negative emotion, neutral emotion, and positive emotion, randomly. The results showed that the young had the highest self-assessment HP under the negative emotion arousal condition, while the old had the highest self-assessment HP under the positive emotion arousal condition. In addition, When both groups were in a positive arousal state, the older group perceived more hazards than the young group. The results could help designers create driving emotions suitable for different driver groups, thus improving their perception of hazards and reducing risky driving.

## Highlights

- Negative emotions increased participants’ self-assessed Hazard Perception.
- Young drivers had the highest perceived hazard in negative emotions, while old drivers had the highest perceived hazard in positive emotions.
- It is important to create the right driving emotion for different driving groups.

## Introduction

Hazard perception (HP) refers to the ability of drivers to detect potential dangers on the road, accurately assess the degree of harm and deal with them (Crundall et al., 2012). Studies show drivers’ demographic characteristics (age), driving behaviors (Song et al., 2021), and driving emotion (Steinhauser et al., 2018; Kadoya et al., 2021b) are crucial in traffic safety. Exploring the relationship between drivers’ demographic characteristics (e.g., age), driving emotions, and dangerous driving behaviors are helpful to reduce property loss and even casualties caused by risky driving.

Research has found that driving style is related to age (Taubman-Ben-Ari et al., 2004, 2016; Poo and Ledesma, 2013; Long and Ruosong, 2019; Navon and Taubman-Ben-Ari, 2019; Padilla et al., 2020), with young drivers like bad driving behavior. Young drivers are prone to be influenced by the external environment in the process of driving, resulting in a series of emotions that affect their cognition, attention, judgment, and behavior, and lead to dangerous driving. Therefore, young drivers are the main group of traffic accidents (Steinhauser et al., 2018; Kadoya et al., 2021a). Although the number of deaths among young drivers has been on the decline (Ferguson et al., 2007), a complete understanding of the potential impact of emotion on dangerous driving is needed.

However, the effect of different emotions on HP in young and old people is still unclear, therefore we studied the cognitive processing of emotions on the HP of different groups of drivers. A comprehensive understanding of how different groups of drivers think and feel about dangerous driving under different driving emotions is helpful to provide theoretical guidance for improving safe driving.

## Related Work

### Hazard Perception

In traffic psychology, HP refers to subjective assessment of the degree of harm caused by traffic accidents, which can affect drivers’ behavioral decisions (Deery, 1999; for example, evaluating dangerous behaviors such as running red lights.). A driver’s perception of danger is affected by driving experience and age. For example, driving experience can improve a driver’s ability to perceive danger (Deery, 1999; Rosenbloom et al., 2008). In addition, some studies believe that negative emotions such as fear and anger can also affect drivers’ perception of danger (Lu et al., 2013).

Some studies have found an antithetical relationship between perceived hazards and dangerous driving behavior. In other words, the stronger the driver’s HP ability is, the less likely he will engage in dangerous driving behavior (Machin and Sankey, 2008; Mills et al., 2008). Besides, the researcher (Ma et al., 2010) further suggested that risk perception may not directly affect drivers’ driving behavior. For example, the researchers used risk perception scales to measure participants’ attitudes toward traffic accidents (for example, how much you care about a traffic accident which might hurt others) and found that perception of danger has an indirect effect on speeding behavior. Moreover, HP moderated the relationship between age, and dangerous driving (Rhodes and Pivik, 2011). In simpler terms, HP is more suitable for predicting dangerous driving in older adults than in young adults.

To sum up, dangerous driving behaviors can be effectively reduced by improving drivers’ danger perception ability, thus improving road driving safety. Therefore, the relationship between HP, affective states, and age needs further study.

### Affective States

Driving emotion (Steinhauser et al., 2018; Kadoya et al., 2021b) is crucial in traffic safety, which can cause dangerous driving behaviors by influencing drivers’ cognition, attention, judgment, and behavior (Steinhauser et al., 2018; Kadoya et al., 2021a). For example, for some drivers who take driving for pleasure, driving itself can be enjoyable and generate positive emotions during driving (Sheller, 2004). Studies have shown that driving in positive emotions, such as letting the guard down and playing with friends, can lead to dangerous driving behavior (Møller and Gregersen, 2008). However, there are opposing views that positive emotions have been found to promote more flexible, creative thinking and action, thus may enhance creativity associated with perceived danger (Isen, 1987; Lyubomirsky et al., 2005).

However, positive emotions are rarely used in road traffic environments and are considered unable to effectively awaken drivers’ awareness of vigilance (Lewis et al., 2007). To keep drivers on high alert in road traffic safety, negative emotions dominated by fear, tension, and worry are often used (Lewis et al., 2008). Obviously, it is important to use negative emotions on the traffic road. Despite this, some studies (Elliott, 2003) suggest that the use of negative emotions should be cautious, because the arousal of fear is stimulating and inhibiting, which may lead to defects in coping mechanisms in road traffic safety. Many studies have found that fear produces adverse reactions (Schoenbachler and Whittler, 1996; Witte et al., 1998; Orit Taubman Ben-Ari and Mikulincer, 2000). This kind of adverse reaction shows that in order to cope with the unpleasant feelings caused by the prompt information, they defensively ignore the prompt information, but do not control or eliminate the potential threat implied in the message. In addition, some people in fear may perceive the prompt as a self-challenge, reducing the perceived level of danger while the actual level of danger remains the same, leading to dangerous driving behavior. Therefore, scholars suggest that negative emotions such as fear should be carefully used in road traffic safety, and other types of emotions should be considered (Wundersitz et al., 2010). Whereas, when people are in a negative emotional state, they may process information more carefully (Schwarz et al., 1991). If warning signals intend to convey information about safety or danger, then negative emotions may promote the cognitive process of danger perception. As a result, it’s not clear which emotions are better for driving.

In addition, there seems to be a gender gap in positive emotions among young drivers: Young male drivers are more enjoy driving than young female drivers, according to a study of driving attitudes (Harré et al., 1996), and male drivers are more affected by positive emotions, which may explain why they are more likely to drive dangerously (Rhodes and Pivik, 2011).

If drivers’ danger perception ability is affected by emotions and age, then road traffic signs should be designed to eliminate and avoid the influence of these factors when conveying potential threat information. The effect of emotion and on the perceived danger of warning messages is unclear. Therefore, it is necessary to further study how young drivers perceive and process warning messages in different emotional states.

### Age Difference

Another issue worth paying attention to is whether age differences exist in people’s processing and response to information under different emotional states. Young drivers (15 to 25 years old) drive unsafe more often than older age groups, research has also found that young drivers’ driving performance is linked to cognition (Di Meco et al., 2021). The elderly have a higher emotional happiness index and less negative emotional experience than young people (Mikels et al., 2005; Reed and Carstensen, 2012; Gronchi et al., 2018). In the process of processing emotional information, the elderly pay more attention to positive emotional information than negative emotional information, that is, the “positive effect” of the elderly. This is also true in memory research (Mather and Carstensen, 2003; Comblain et al., 2005; Spaniol et al., 2008; Scheibe and Carstensen, 2010).

Two models of cognitive-affective aging: social-emotional selectivity theory (Carstensen et al., 1999; Mikels et al., 2005) and dynamic integration theory (Labouvie-Vief, 2003, 2005, 2009; Labouvie-Vief et al., 2009) explain the positive effects.

On the one hand, SST is a life-span development theory related to motivation (Mather and Carstensen, 2003), assuming that goal selection follows adulthood. SST proposes a choice of goals throughout adulthood. Some goals involve preparing for the future, such as accumulating knowledge and meeting new friends; Other goals are related to satisfying the present life, such as enjoying intimacy and pursuing emotional satisfaction. While these goals are important over the course of life, SST places great emphasis on the negative correlation between age and future time horizons. Young people tend to perceive that the future is vast, prioritizing preparation for the future, spending more time and energy building social networks and increasing knowledge reserves to cope with an uncertain future; older adults seek to meet the emotional satisfaction of the present when they perceive the limited time in the future (Sims et al., 2015). However, when the elderly are getting older or perceive that time in the future is limited, the pursuit of emotional satisfaction takes precedence over other goals (Sims et al., 2015). The positive effect theory of SST extension theory proposes that the larger the age span of the two study groups, the easier the positive effect will be detected (Charles and Hong, 2015).

On the other hand, DIT suggests that this is an automatic process of cognitive decline due to aging (Labouvie-Vief, 2003). This theory suggests that older adults have difficulty managing cognitive-affective complexity due to the limitations of age-related cognitive resources. The theory believe that positive information is easier than negative information, and older people use fewer cognitive resources to process positive information. The elderly face the decline of cognitive resources and thus compensate for the processing of information in a simple, positive way, that is, the automatic processing of positive emotional information. Automatic processing of positive emotional information is an adaptive attentional mechanism (Labouvie-Vief, 2009), when it is associated with negative emotions such as pain and threat, it automatically preserves cognitive processing by eliminating emotional stimuli. Therefore, the positive effect of emotion processing on the elderly is minimal automatic emotion processing. The above studies showed that older people prefer positive emotional processing, while younger people do not have this preference or prefer negative emotional processing (Kennedy et al., 2004).

Both SST and DIT explain differences in emotional processing in the elderly. Positive information processing stabilized or improved with age, while negative information processing decreased with age (Carstensen et al., 1999; Labouvie-Vief, 2003). While the role of attention in each theory may lead to different assumptions. SST believes that the positive effect depends on the later attention process, which is passively controlled, while DIT believes that the positive effect depends on the early attention process, which is processed automatically.

Studies have shown that increased attention to negative material among young people leads to age-related differences in the direction of attention (Charles et al., 2003; Ready et al., 2007; Shamaskin et al., 2010). And the elderly showed a stimulative effect of attention on positive (as opposed to negative) information (Isaacowitz et al., 2006; Mather and Knight, 2006).

In conclusion, young drivers and old drivers prefer different emotional information (positive or negative), however, different emotional stimuli will guide older drivers and young drivers to judge and make decisions differently (Taubman-Ben-Ari, 2012). What is more, the influence of emotion on HP of young and old drivers is still unclear. Therefore, it is necessary to further study the influence and effect of emotion on different driving groups.

### The Present Work

At present, studies on dangerous driving mainly focus on young drivers and adult drivers, but as China continues to enter an aging society, there are more and more older drivers. Therefore, We need to further explore the HP ability of young and old drivers in different emotional states. Therefore, this study explores the following questions:

1. Do emotions have a significant influence on drivers’ perceived danger?
2. Are there differences in perceived hazard between young and old drivers under different emotional states?

In this study, we used the method of listening to music while recalling imagination (Kuhbandner and Zehetleitner, 2011; Larson et al., 2013; van Steenbergen et al., 2014) to wake up the emotions of the participants. The Self-Assessment Manikin (SAM; Morris, 1995) questionnaire was used to assess their emotional valence (EV) and emotional arousal (EA), and evaluated their emotional state at that time from these two dimensions. A five-point scale questionnaire (Kalsher et al., 1995) was adopted to ask the participants to score the HP of 22 traffic warning signs. We hypothesized that emotions have a significant impact on drivers’ HP, and drivers’ HP is higher under negative emotions; the influence of age and emotion on HP is interactive, young people have higher HP under negative emotion, while old people have higher HP under positive emotion.

## Materials and Methods

### Design

**FIGURE 1:**
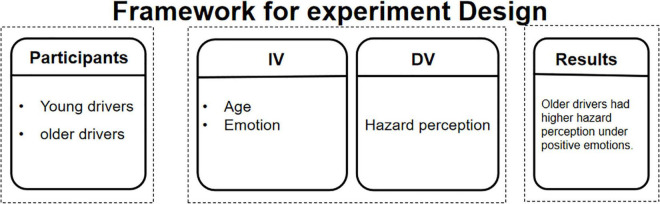
Framework for experiment design.

We designed a 3 (emotion: Negative, Neutral, and Positive) × 2 (age: young group, old group) mixed experimental design. Emotion and age are independent variables, the former is a within-participants variable and the latter is a between-participants factor. The dependent variable was the self-assessment of the HP of traffic warning signs. The framework for experimental design is shown in Figure 1.

### Participants

We recruited 14 young drivers with an age range of 21 to 24 (*M* = 22.21, SD = 1.05) and 13 older drivers with an age range of 50 to 60 (*M* = 54.08, SD = 2.72). There were nine men and five women among the younger drivers, and twelve men and one woman among the older drivers. The young drivers are undergraduate students from Ningbo University. The older drivers are didi ‘s express drivers (Non-professional driver), as long as they meet the application requirements (age: 22–60; Driver’s license C2 or above for express; Driving experience more than 1 year; and Drivers with a total mileage of less than 100,000 km) can apply through Didi’s express drivers. These drivers usually pick up customers on the way home and earn part of the fee, which does not have the same positive feelings toward customers as professional drivers.

In order to control the driver’s driving experience at a similar level, we first measure the driver’s driving activity level (driving age, weekly driving frequency, driving mileage, and the number of traffic accidents) through questionnaires. Drivers with driving experience of more than 1 year and less than 2 years, driving frequency of three times or more per week, total mileage of 10,000 or so, and no traffic accidents were selected and invited as participants to participate in the experiment, so as to avoid the influence of driving experience as the intervention variable. The gender composition of the two groups was not deliberately controlled, but the result of random invitations. All participants had normal or corrected visual acuity and were not color blind.

According to the mixed experimental design, we randomly divided participants into three (Negative, Neutral, and Positive) emotion groups, repeatedly arousing three different emotional states over different time periods. Each participant completed the experimental tasks measured by the questionnaire in three different emotional states.

G* Power (Faul et al., 2007) was used for prior power analysis. Under the conditions of standard effect size f is 0.4 (large), significance level of 5% and power of 80%, the total sample size required was 64. Therefore, the minimum sample size of young (old) drivers should be 11.

### Materials

**FIGURE 2:**
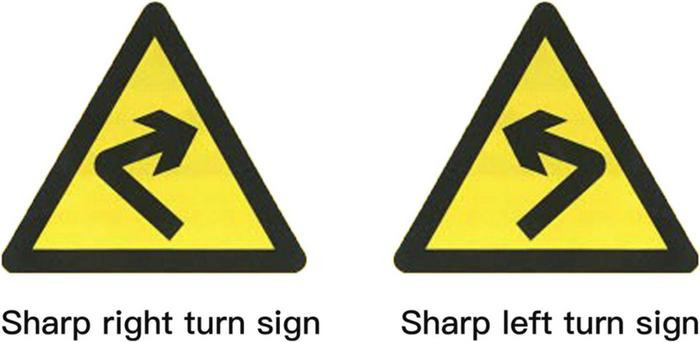
Screening criteria: the same meaning but opposite direction of the sign to choose one, such as sharp left turn and sharp right turn.

To exclude the influence of incomprehensibility on the participants’ self-assessment of perceived hazard. We measured the understanding of Chinese road traffic warning signs stipulated in Road Traffic Signs and Lines (GB5768) and selected the signs with an understandable level of 70% as experimental materials for the questionnaire. In order to avoid and reduce the weariness caused by too many questions in the traffic warning sign comprehension experiment, we randomly selected one of the signs with opposite directions but the same meaning. For example, “turn left” or “turn right” were randomly selected as test signs in the traffic sign comprehension experiment, as shown in Figure 2. Participants would see one of the signs and fill in its meaning in the experiment. This preliminary screening method can not only avoid subjective exclusion but also reduce the number of questions to a certain extent.

**FIGURE 3:**
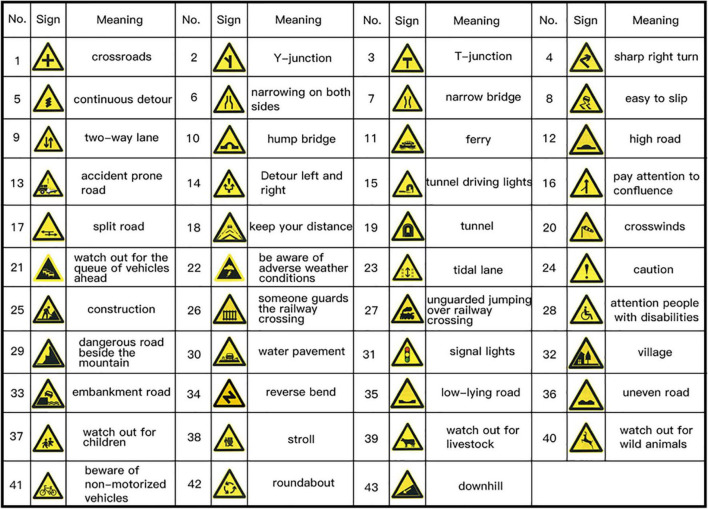
Preliminary screening of 43 traffic warning signs.

Finally we selected 43 triangular warning signs (as shown in Figure 3) from the 77 road traffic warning signs stipulated in Road Traffic Signs and Lines (GB5768) as the test signs of the experiment.

Remove the text message of the 43 signs, and only the logo image was retained and printed in color on A4 white paper. Each page had 6 signs with a size of 2 cm × 2 cm. At the top of the questionnaire, we used a description of the “Yield pedestrian” sign as a reference.

We invited 10 people (including inexperienced, novice, and skilled drivers) to participate in the traffic warning signs comprehension experiment. The participants ranged from 21 to 45 years old (*M* = 25.4, SD = 6.98). All participants had normal or corrected visual acuity, were not color blind, did not participated in similar experiments recently.

After simply informing participants of the purpose and process of the experiment, we asked them to write down their understanding meanings under the 43 traffic warning signs on the paper questionnaire as detailed as possible. The experiment lasted about 30 min.

To evaluate the fairness, two independent raters gave corresponding comprehension scores for the meaning description written by the participants according to the scoring criteria of the open questionnaire scale, as shown in Table 1. Cronbach’s alpha coefficient value of the scale was 0.96, indicating high reliability of the scale (Duarte and Rebelo, 2005).

**TABLE 1:** Scoring criteria sacle.

Type	Specific meaning	Score
1	The accurate understanding of the sign is over 80%.	1
2	The accurate understanding of the sign is 66 ∼ 80%.	0.75
3	The accurate understanding of the sign is 50 ∼ 65%	0.5
4	The answer given is “I don’t know.”	0 –1
5	The answer given contradicts the meaning conveyed by the sign.	–1

Descriptive statistics of the comprehensible rate of 43 signs are shown in Table 2.

**TABLE 2:** Descriptive statistics of the comprehension rate of 43 signs.

No.	Meaning	Comprehension rate (%)
1	Crossroads	100.00
2	Y-junction	90.00
3	T-junction	65.00
4	Sharp right turn	70.00
5	Continuous detour	100.00
6	Narrowing on both sides	27.50
7	Narrow bridge	22.50
8	Easy to slip	77.50
9	Two-way lane	85.00
10	Hump bridge	20.00
11	Ferry	60.00
12	High road	75.00
13	Accident prone road	77.50
14	Detour left and right	32.50
15	Tunnel driving lights	80.00
16	Pay attention to confluence	82.50
17	Split road	–40.00
18	Keep your distance	50.00
19	Tunnel	100.00
20	Crosswinds	50.00
21	Watch out for the queue of vehicles ahead	2.50
22	Be aware of adverse weather conditions	70.00
23	Tidal lane	–40.00
24	Caution	97.50
25	Construction	97.50
26	Someone guards the railway crossing	–12.50
27	Unguarded jumping over railway crossing	77.50
28	Attention people with disabilities	47.50
29	Dangerous road beside the mountain	40.00
30	Water pavement	85.00
31	Signal lights	100.00
32	Village	87.50
33	Embankment road	82.50
34	Reverse bend	65.00
35	Low-lying road	85.00
36	Uneven road	75.00
37	Watch out for children	75.00
38	Stroll	100.00
39	Watch out for livestock	85.00
40	Watch out for wild animals	80.00
41	Beware of non-motorized vehicles	62.50
42	Roundabout	87.50
43	Downhill	60.00
1–43	All Signs	60.00

**FIGURE 4:**
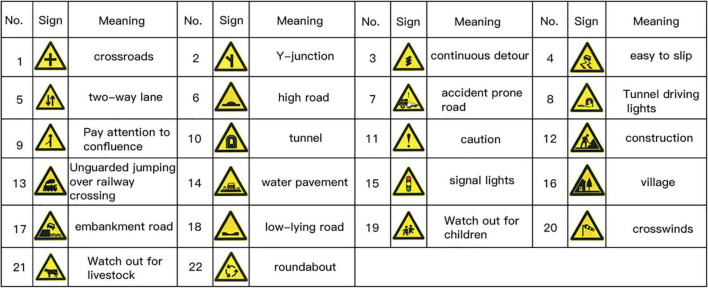
22 traffic warning signs with an understanding rate of 67% or more.

The understanding rates for all signs ranged from −40.0% (Sign 17 — split road) to 100% (Sign 1 — crossroads), with an overall mean (±standard deviation) of 60.0% (±25.83%) understanding rates for all signs. Ten signs had an understanding rate of greater than 85% as recommended by ANSI Z535.3, 2002, and 15 signs had an understanding rate of between 67 and 85% that is considered acceptable by ISO 3864-3, 2006 (a sign understanding rate of greater than 67%). Among them, the “low-lying road” sign, “high-protrusion road” sign, and “uneven road” sign have similar meanings, so the low-lying road sign with the highest understanding rate is retained. Although the understanding rate of the “Watch out for wild animals” sign reaches 80.0%, it is not common in daily traffic roads, so we removed it. After the sign comprehension test, we screened out 22 road traffic warning signs meeting the requirements, as shown in Figure 4.

### Procedure

#### Pre-experiments

We conducted a preliminary experiment to test whether listening to music and recalling the past successfully aroused the corresponding emotional state in participants. In this study, we used the method of listening to music while recalling and imagining (Kuhbandner and Zehetleitner, 2011; Larson et al., 2013; van Steenbergen et al., 2014) to induce the emotions of the participants.

We invited another 20 students (10 males) from Ningbo University to participate in the preliminary experiment. The pre-experiments are conducted in quiet, independent laboratories. Each participant was asked to complete two EA (negative and positive) tests at random. After a brief explanation of the purpose and procedure of the experiment, we played the corresponding musical selections to the participants (Jefferies et al., 2008), and the participants were asked to listen to music while recalling past events which had made them very delighted or very depressed, it took about 10 min. In order to avoid the boredom caused by repeated listening to a song in the group, each emotion group selected two songs to play in turn. We played “Uranus, The Magician, The Planets” and “Mars, The Bringer of War, The Planets” by Holst for the negative emotion group; Mozart’s “Eine Kleine Nachtmusik: Rondo” and “Eine Kleine Nachtmusik: Allegro” for the positive emotion group.

We adopted the SAM scale (Morris, 1995) to measure each participant’ emotional state which is a 9-point scale. Each participant was asked to score at their current EV from “1” to “9” (1 = “unhappy,” 9 = happy), and their current EA from “1” to “9” (1 = “I did not feel any stimulus,” 9 = “I felt a strong stimulus”).

After the EA test, we used SPSS 19.0 data analysis software to conduct one-way ANOVA on the data of EV and EA of participants in different emotional groups.

After data analysis, the mean EV of the positive emotion group was 6.50, SD = 0.76, and mean EA was 5.35, SD = 1.98. The mean EV of the negative emotion group was 4.00, SD = 0.80, and mean EA was 4.50, SD = 1.40. Results showed that the EV of the positive emotion group was significantly higher than that of the negative group (*P* = 0.008). While there was no significant difference between the EA (*P* = 0.125), indicating that participants in both groups had similar levels of arousal and listening to music and recalling the past induced the corresponding emotional state successfully.

#### Formal Experiment

After the preliminary experiment, we carried out the formal experiment. Each participant entered three stages of random arousal (negative, neutral, and positive), respectively. Before the formal experiment began, all participants (14 young drivers, 13 older drivers) were asked to read and sign an informed consent form. They were then simply told that the purpose of the music was to arouse their emotions.

First of all, the negative emotion group and the neutral emotion group were awakened the corresponding emotions, respectively, while the neutral emotion group did not awaken the emotion. The process of EA was consistent with the pre-experiment. We recorded the EV and EA before the HP test as EV1 and EA1, respectively.

In addition, 22 traffic warning signs are printed in color on A4 white paper, each with a related question: Please tick “√” where the hazard level of this sign corresponds to your understanding. All participants used a five-point scale (Kalsher et al., 1995) to score the perceived danger of 22 traffic warning signs, with “1” representing no danger at all and “5” representing fatal. The music continued throughout the experiment until it was all over. After the participants completed the HP questionnaire, using the SAM scale to evaluate the immediate emotional valence (EV2) and emotional arousal (EA2) level again.

The young drivers completed the experiment in a quiet and independent laboratory in the school. In order to avoid the tension of the elderly drivers due to the strange environment, we asked the elderly drivers to sit in the main driving position of their didi car, keep the car stationary, let the host sitting in the back read out the questions, and record their oral scoring.

Every participant’s emotional state was randomly awakened to eliminate the effect of the experiment’s order, and the experiment lasted about 40 to 50 min for each participant. Before the participants’ first EA experiment began, we first proceeded by asking questions to understand the emotional state of each participant, so as to awaken the corresponding emotional state by the way, and after each EA and HP questionnaire was completed, we would chat with the participant for about 20 min to soothe the participant’s emotions and eliminate the influence of the previous emotional manipulation, in preparation for the next EA. The second EA is a random awakening of a certain emotion, so there are participants who are awakened to negative emotions in the third EA, but we will adjust the participants’ emotions to normal levels through chat after the experiment is over.

After completing the experiment, each participant received a cash reward of 20 yuan RMB. Finally, we calculated the self-assessment HP scores of all the 22 traffic warning signs.

### Data Acquisition

The participants data were emotional state data from the SAM scale and self-rated HP data from the HP questionnaire. 6 questionnaire data from the young drivers and 3 questionnaire data from the old drivers were excluded from the analysis due to the significant difference in the EV of the participants before and after the experiment. We used SPSS 19.0 data analysis software to analyze the data of all participants.

### Emotional Manipulation

One-way analysis of variance was used to compare the differences in EV and EA scores between different emotional groups, so as to judge whether the emotions of the subjects were successfully aroused.

In the emotion data before starting to fill out the HP questionnaire score (EV 1and EA1), emotion was significant for EV1 score [(*F* (2,69) = 247.54, *p* < 0.001)] and EA1 score [(*F* (2,69) = 9.40, *p* < 0.001)]. *Post hoc* Comparison analysis showed that the EV1 score of the negative emotion group (*M* = 2.08, SD = 1.06) was significantly lower (*p* < 0.001) than those of the neutral emotion group (*M* = 5.67, SD = 0.71), and also significantly lower (*p* < 0.001) than those of the positive emotion group (*M* = 7.38, SD = 0.71). The EV1 scores of participants in the neutral emotion group were significantly lower (*p* < 0.001) than those in the positive emotion group.

Similarly, the EA1 scores of the negative emotion group (*M* = 5.12, SD = 1.12) were significantly lower (*p* < 0.001) than those of the positive emotion group (*M* = 6.42, SD = 1.14), and also significantly lower (*p* < 0.001) than the neutral emotion group (*M* = 6.25, SD = 1.11). However, there was no significant difference in EAl score between neutral emotion group and positive emotion group (*p* > 0.05), indicating that all participants were in a state of high arousal. These results confirm that we successfully evoked the corresponding emotional states in each group of participants.

In the emotion data after filling out the HP questionnaire (EV2 and EA2), emotion had significant effect on the EV2 score [*F* (2,69) = 152.91, *p* < 0.001], and the *post hoc* analysis showed that the EV2 score of the negative emotion group (*M* = 2.63, SD = 1.01) was significantly lower (*p* < 0.001) than that of the neutral emotion group (*M* = 5.33, SD = 0.76), and also significantly lower (*p* < 0.001) than that of the positive emotion group (*M* = 7.13, SD = 0.90). The EV2 scores of participants in the neutral emotion group were significantly lower than those in the positive emotion group (*p* < 0.001). But the EA2 score [*F* (2,69) = 0.87, *p* > 0.05] was not significant. It showed that after the completion of the test task, although the arousal degree of the three groups was at a medium level, the participants still maintained the corresponding emotional state.

## Results

The commonly used data analysis method for subjective evaluation is to calculate the average value, that is, to calculate the average HP score after adding the scores corresponding to each traffic warning sign on the HP questionnaire. Self-assessment HP of 22 tested road traffic warning signs was collected, and descriptive statistics were conducted on the score of HP of sample signs with SPSS 19.0 software. Table 3 shows the statistical results.

**TABLE 3:** HP scores in different conditions (age × emotion).

Age	Emotion	Statistics	HP score
Young	Negative	*M*	3.39
		SD	0.31
	Neutral	*M*	2.71
		SD	0.29
	Positive	*M*	2.76
		SD	0.71
Old	Negative	*M*	3.02
		SD	0.43
	Neutral	*M*	2.80
		SD	0.49
	Positive	*M*	3.55
		SD	0.49

If the main effect of emotion or age was significant in the analysis of variance, the differences among each group were further analyzed by *post hoc*.

### Influence of Emotion on Hazard Perception

The effects of emotion and age on self-assessed HP were analyzed using two-factor repeated variance measures. The results of two-factor repeated variance measures shown below: Firstly, the main effect of emotion was significant [*F* (2,66) = 6.520, *p* = 0.003], which means that emotion had a significant effect on self-assessed HP. Further analysis showed that the negative emotion group (*M* = 3.20, SD = 0.10) perceived more harm (*p* = 0.002) than the neutral emotion group (*M* = 2.75, SD = 0.10), and the positive emotion group (*M* = 3.15, SD = 0.10) also perceived more harm (*p* = 0.005) than the neutral emotion group. In other words, in the neutral emotional state, the participants perceived the lowest perceived hazards. Results of paired comparison of emotions analysis are shown in Table 4.

**TABLE 4:** Results of paired comparison of emotions analysis.

(I) Emotion	(J) Emotion	Mean difference (I-J)	Standard error (SE)	*P*	95% confidence interval	
					Lower bound	Upper bound
Neutral	Negative	−0.449*	0.136	0.002	−0.721	−0.177
	Positive	−0.400*	0.136	0.005	−0.672	−0.127

*Based on the estimated marginal mean.*

*The mean difference was significant at 0.05 level.

### Influence of Age and Emotion on Hazard Perception

The main effect of age was not significant, age had no significant effect on self-assessed HP, but the interaction effect of emotion and age was significant [*F* (2,66) = 9.18, *p* < 0.001]. Furthermore, simple effect analysis showed that the self-assessed HP of the young group was higher under negative emotion than under neutral emotion (*p* = 0.001) and positive emotion arousal (*p* = 0.002). In other words, the self-assessed HP of the young group was the highest under negative EA. For another, elderly group participants under the condition of positive emotion awaken was higher than in negative emotion awakened self-assessment HP (*p* = 0.008) and neutral emotion awaken self-assessment HP (*p* < 0.001). In other words, the older group participants had the highest HP in self-assessment under positive emotional conditions. Finally, when both groups were in positive EA, younger drivers perceived significantly fewer hazards than older drivers (*p* < 0.001). Results of simple effect analysis are shown in Table 5.

**TABLE 5:** Results of simple effect analysis (age × emotion).

Age	(I) Emotion	(J) Emotion	Mean difference (I-J)	Standard error (SE)	*P*	95% confidence interval	
						Lower bound	Upper bound
Young	Negative	Neutral	0.674*	0.193	0.001	0.289	1.059
	Negative	Positive	0.629*	0.193	0.002	0.244	1.014
	Neutral	Positive	−0.045	0.193	0.814	−0.430	0.340
Old	Negative	Neutral	0.223	0.193	0.251	−0.161	0.608
	Positive	Negative	0.530*	0.193	0.008	0.145	0.915
	Positive	Neutral	0.754*	0.193	0.000	0.369	1.139

*Based on the estimated marginal mean.*

*The mean difference was significant at 0.05 level.

## Discussion

### The Influence of Emotion on Self-Assessed Hazard Perception

Emotion of the participants’ self-assessment HP research results show that emotion significantly influences participants’ self-assessment of HP. Further analysis found that the negative and positive emotion groups are perceived more harm than the neutral emotion group, in other words, participants had the lowest HP under neutral emotion. Emotion and gender of the participant’s self-assessment HP research results show that both the neutral and positive emotion groups perceived less harm than the negative emotion group. In other words, participants in the negative emotional group perceived the highest levels of HP. The results of both indicated that negative emotions increased participants’ self-assessed HP.

Excluding the effect of sign familiarity, this may be because negative emotions may encourage people to process information more carefully (Schwarz et al., 1991). If warning signals intend to convey information about safety or danger, then negative emotions may promote the cognitive process of perceived danger. When the drivers in negative emotional conditions saw these traffic warning signs, the negative emotional condition might encourage them to observe the information conveyed by the warning signs more carefully and focus most of their attention on the details of the related HP. This may explain why participants perceived the most danger under negative emotions.

### The Interaction Between Emotion and Age on Self-Assessed Hazard Perception

Under the negative emotion arousal condition, the younger group perceived more harm than the older group, while under the positive emotion arousal condition, the older group perceived more harm than the younger group. This may be because young people have a negative bias in emotional selection while older people have a positive bias (Gronchi et al., 2018). When the young group was in the arousal state of positive emotions, they might avoid positive emotions and inhibit the processing of positive warning signs in the visual processing stage. However, when young people see these traffic warning signs in the state of negative EA, they will focus on the information of negative warning signs, thus generating attention and memory, and negative emotions may encourage people to be more careful in information processing (Schwarz et al., 1991), and focus most of the attention on the relevant HP details (Clore and Palmer, 2009).

The “positive warning sign” here refers to the fact that if a warning traffic sign is designed to convey positive emotions, this may potentially affect the young driver’s perception of danger. For example, a traffic warning sign used to convey the accident-prone road ahead is depicted as a warm home picture, rather than a serious impact and rear-end collision. This can be called a positive warning sign and may have a revelatory effect on the design and application of road traffic warning signs for older drivers.

Dynamic integration theory holds that emotions and cognition are related. According to this theory, positive information is more straightforward than negative information, and people will use fewer cognitive resources to process positive information. Compared with young people, older people will have reduced cognitive resources. The cognitive load capacity decreases and compensates for the preference of positive emotion processing and receiving information and evaluates external objects simply and positively, that is, automatic processing of positive emotion information (Labouvie-Vief, 2009). While positive emotions could influence people to think, feel, and act creatively (Lyubomirsky et al., 2005). When the elderly drivers were in a positive emotional state and saw these traffic warning signs, creativity associated with danger perception may be improved (Isen, 1987). This may reasonably explain why young drivers with negative emotions have higher HP of road traffic warning signs, while older drivers with positive emotions have higher HP of road traffic warning signs.

## Conclusion

In this experimental study, emotion and age have a significant interaction effect on drivers’ self-assessed HP, specifically, young drivers have a higher HP under negative emotions, while older drivers have a higher HP under positive emotions. The results emphasize that driver emotion is an important factor affecting HP, and creating positive emotion in the driving environment is more suitable for elderly drivers. It can be considered to create appropriate driving emotions for different driver groups, to help improve the **HP** of drivers.

It is worth noting the age range of older drivers in this study is 50 to 60 years old (*M* = 54.08, SD = 2.72), which does not meet the standard age for the elderly specified in China (60 years old). However, the age of Didi drivers is less than 60 years old in China, and the cognitive load and understanding ability of 50–60 years old drivers are very different, so it is worth studying the aged drivers in this age group. That’s why we chose 50–60 year old drivers as participants. The age range of 50–60 (*M* = 54.08, SD = 2.72) may can be reasonably considered as the elderly among the drivers compared with young drivers (*M* = 22.21, SD = 1.05).

However, there are still some limitations in this study: First, the perceived effect of road warning signs presented in paper questionnaires is different from that in real driving scenes. Secondly, although the marks selected in this study have been evaluated through the experiment to exclude the influence of comprehension since these participants are not the same as those in the formal experiment, it is difficult for us to confirm that all participants in the formal experiment have correctly understood their meanings, which may be a deficiency. Finally, relevant studies show that driving experience is of great significance to drivers’ perception of danger, and more driving indicators can be measured in the future, to conduct a more comprehensive discussion on the perception of hazard.

## Data Availability Statement

The original contributions presented in the study are included in the article/Supplementary Material, further inquiries can be directed to the corresponding authors.

## Ethics Statement

Written informed consent was obtained from the individual(s) for the publication of any potentially identifiable images or data included in this article.

## Author Contributions

FH conceived the experimental process and invited experimental subjects. RG proposed research topics, conducted experiments, and wrote most of the manuscripts. CS conducted data analysis. GH reviewed the draft and made revisions. All authors edited the manuscript.

## Conflict of Interest

The authors declare that the research was conducted in the absence of any commercial or financial relationships that could be construed as a potential conflict of interest.

## Publisher’s Note

All claims expressed in this article are solely those of the authors and do not necessarily represent those of their affiliated organizations, or those of the publisher, the editors and the reviewers. Any product that may be evaluated in this article, or claim that may be made by its manufacturer, is not guaranteed or endorsed by the publisher.
