# Supplementary figures and images for: Age Differences in Hazard Perception of Drivers: The Roles of Emotion
Source: Front Psychol. 2022 Jun 2;13:867673. doi: 10.3389/fpsyg.2022.867673 (PMC9200974; doi:10.3389/fpsyg.2022.867673)

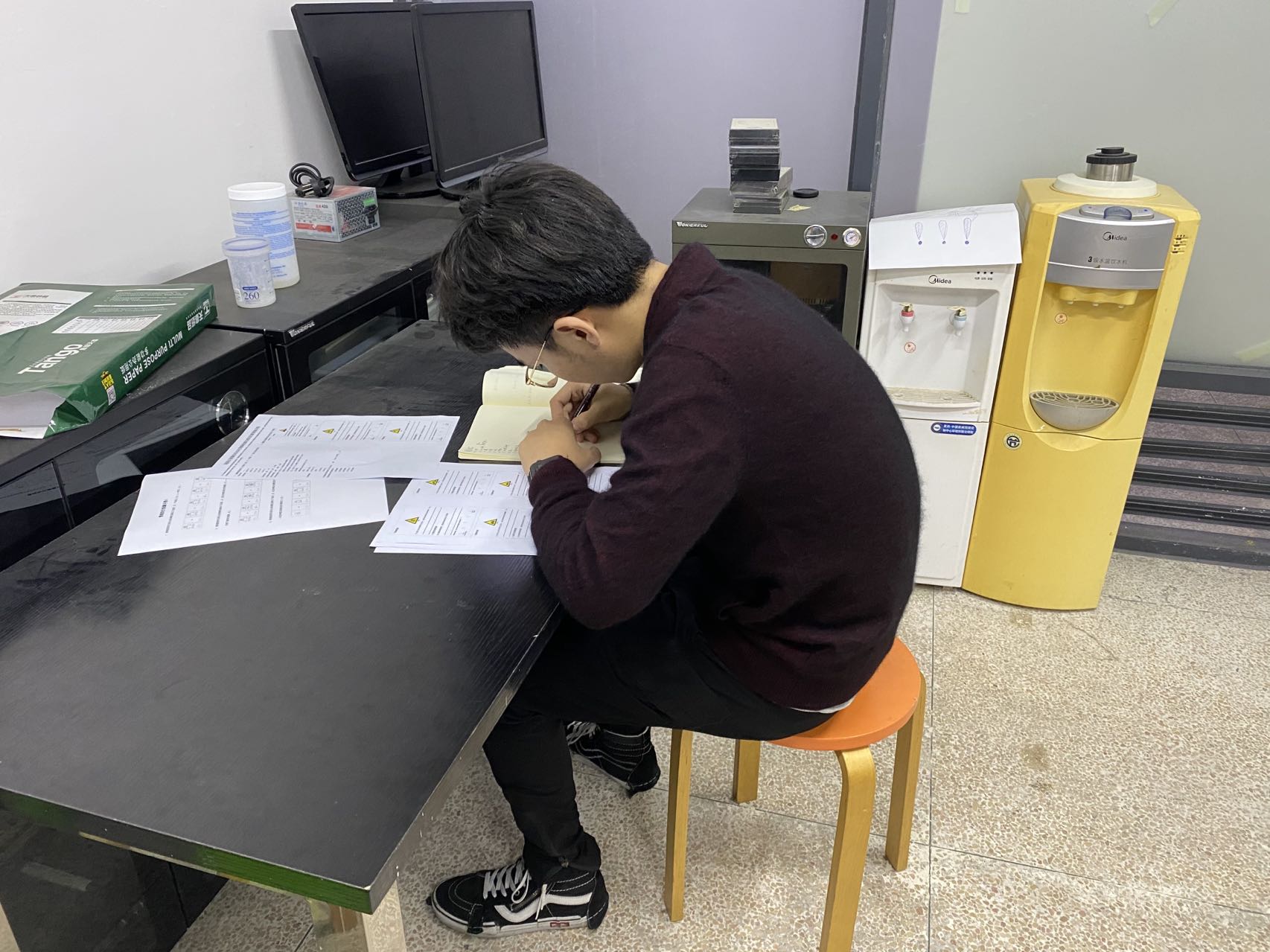

Supplement: Supplementary file 1 [file Data_Sheet_1.ZIP › 120849be979c44bb5e130bf696ab310.jpg]

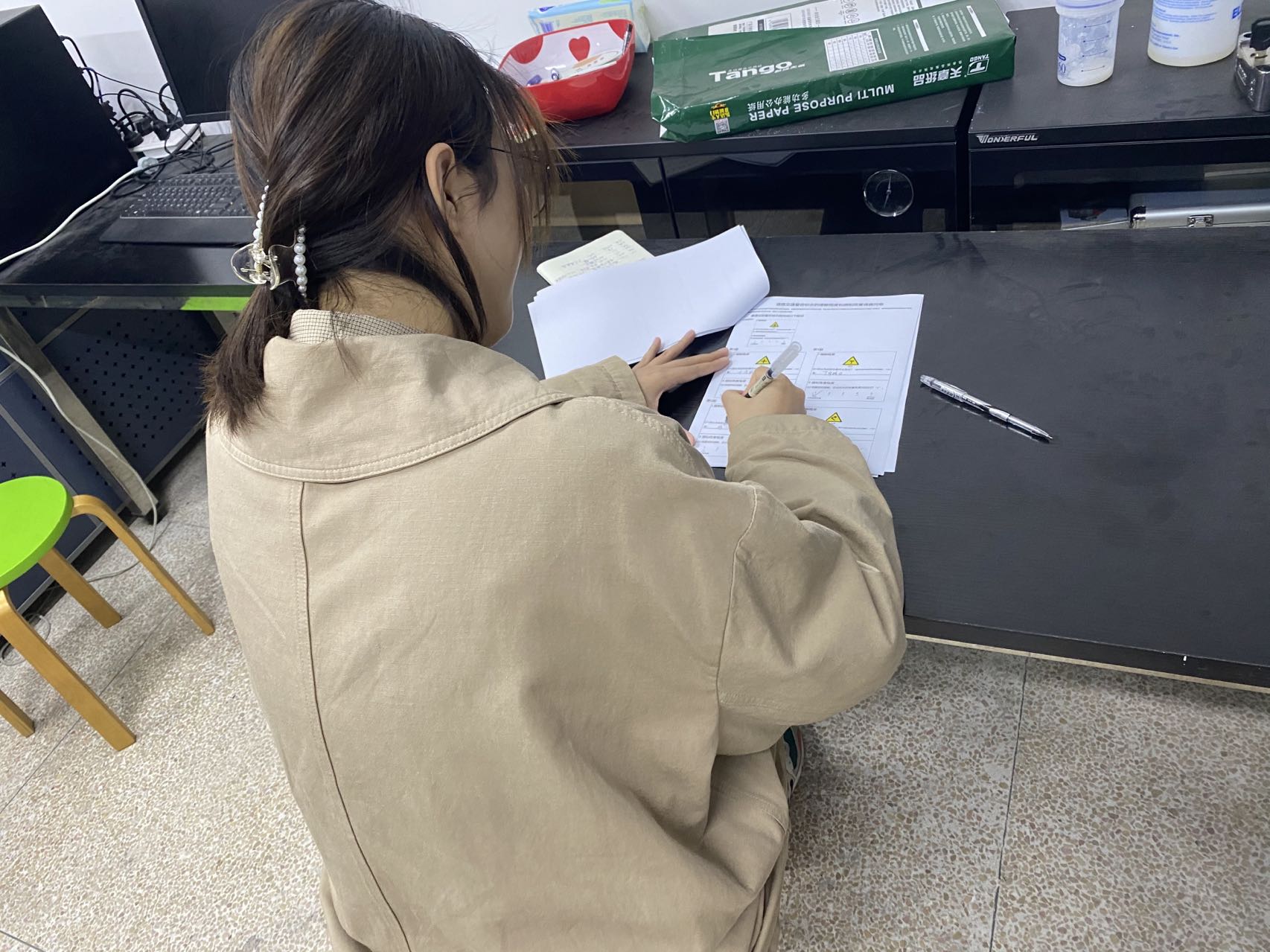

Supplement: Supplementary file 1 [file Data_Sheet_1.ZIP › 4c06103366970167becb8fe5c0396f1.jpg]

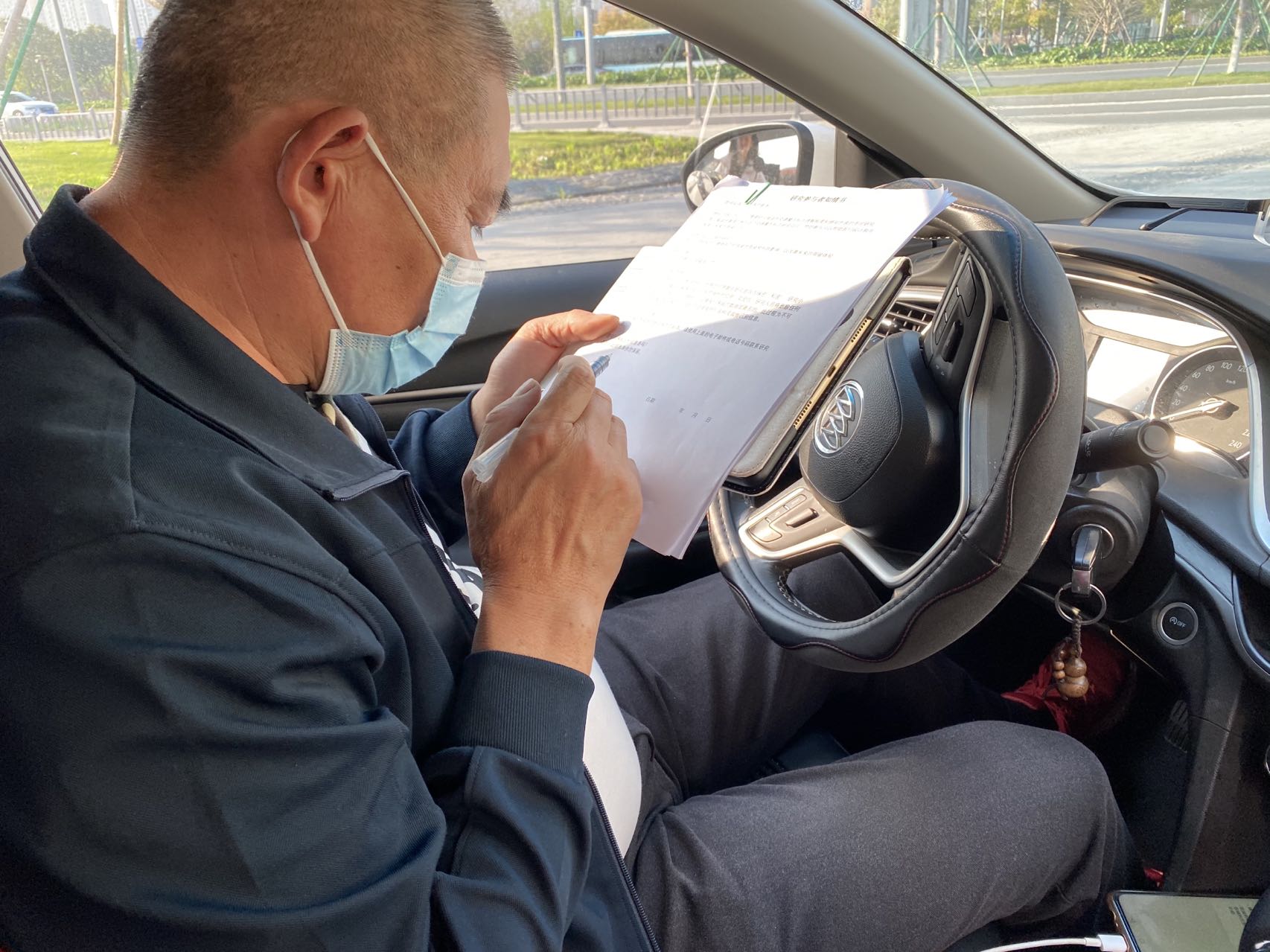

Supplement: Supplementary file 1 [file Data_Sheet_1.ZIP › 6677af57b36e4f6ce2c7b363b238bc3.jpg]

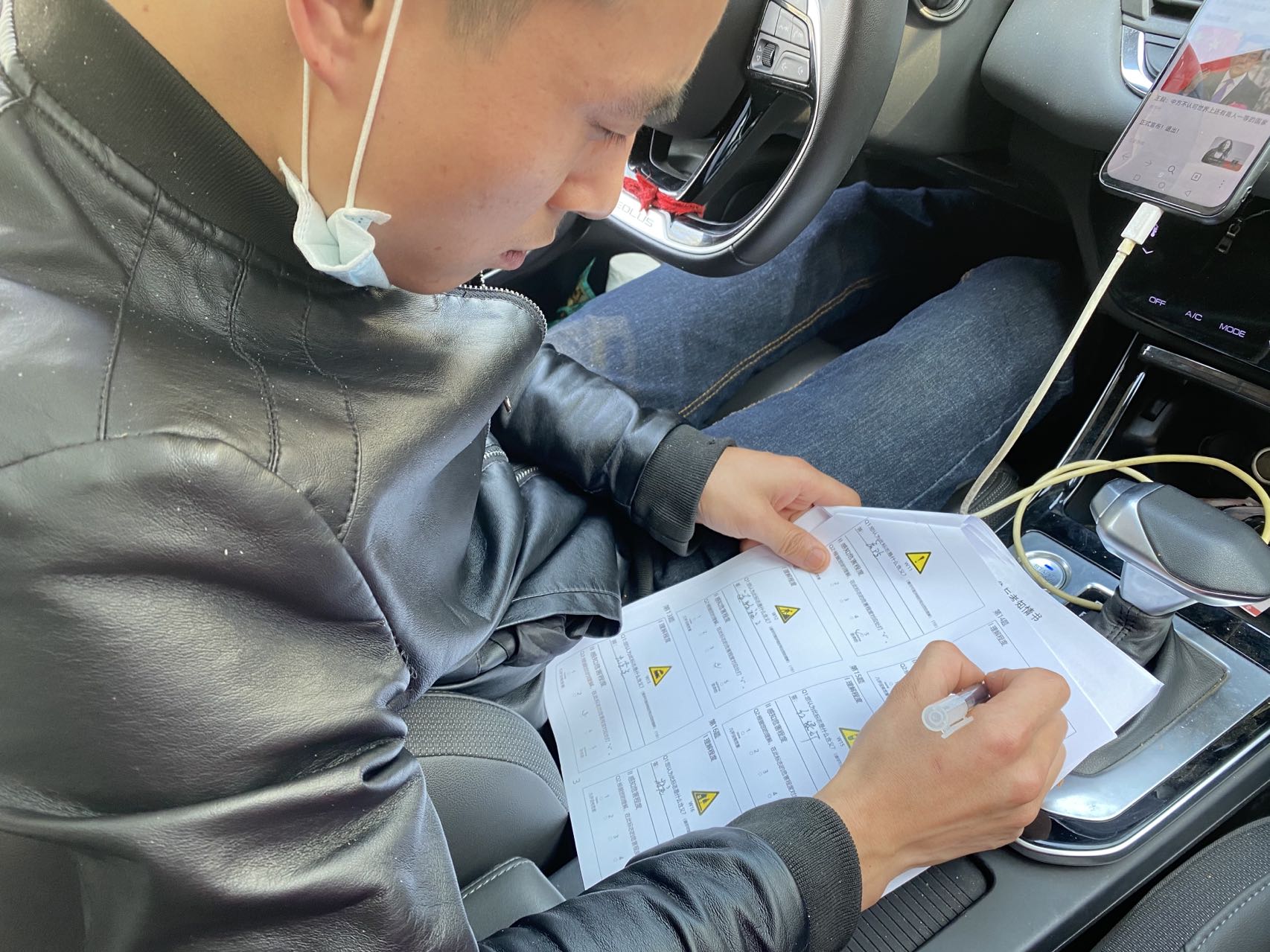

Supplement: Supplementary file 1 [file Data_Sheet_1.ZIP › 836f300599a7934a0ac9ef192a46452.jpg]

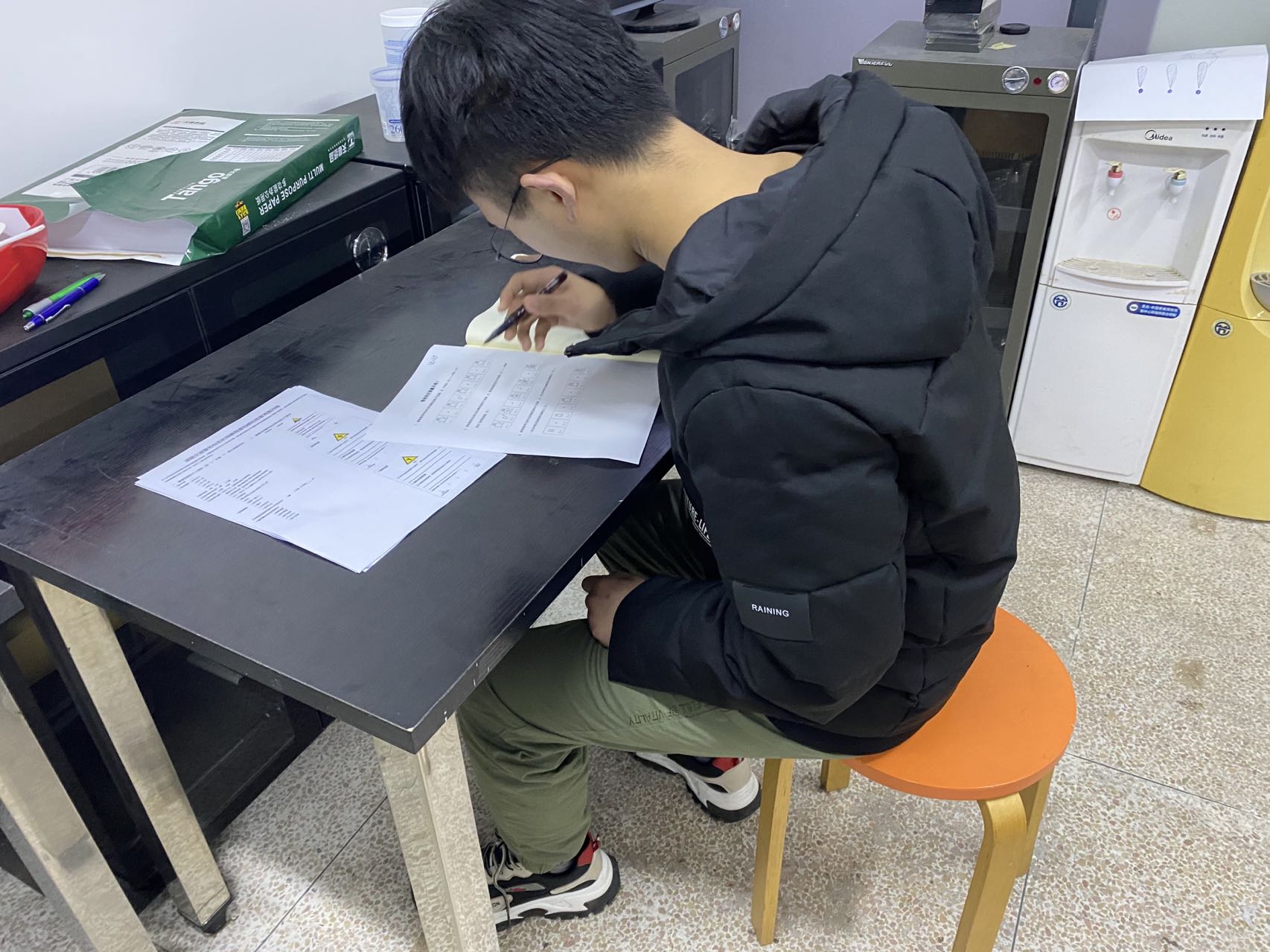

Supplement: Supplementary file 1 [file Data_Sheet_1.ZIP › 9d36cdfab07180e670ed8e44dd5d741.jpg]

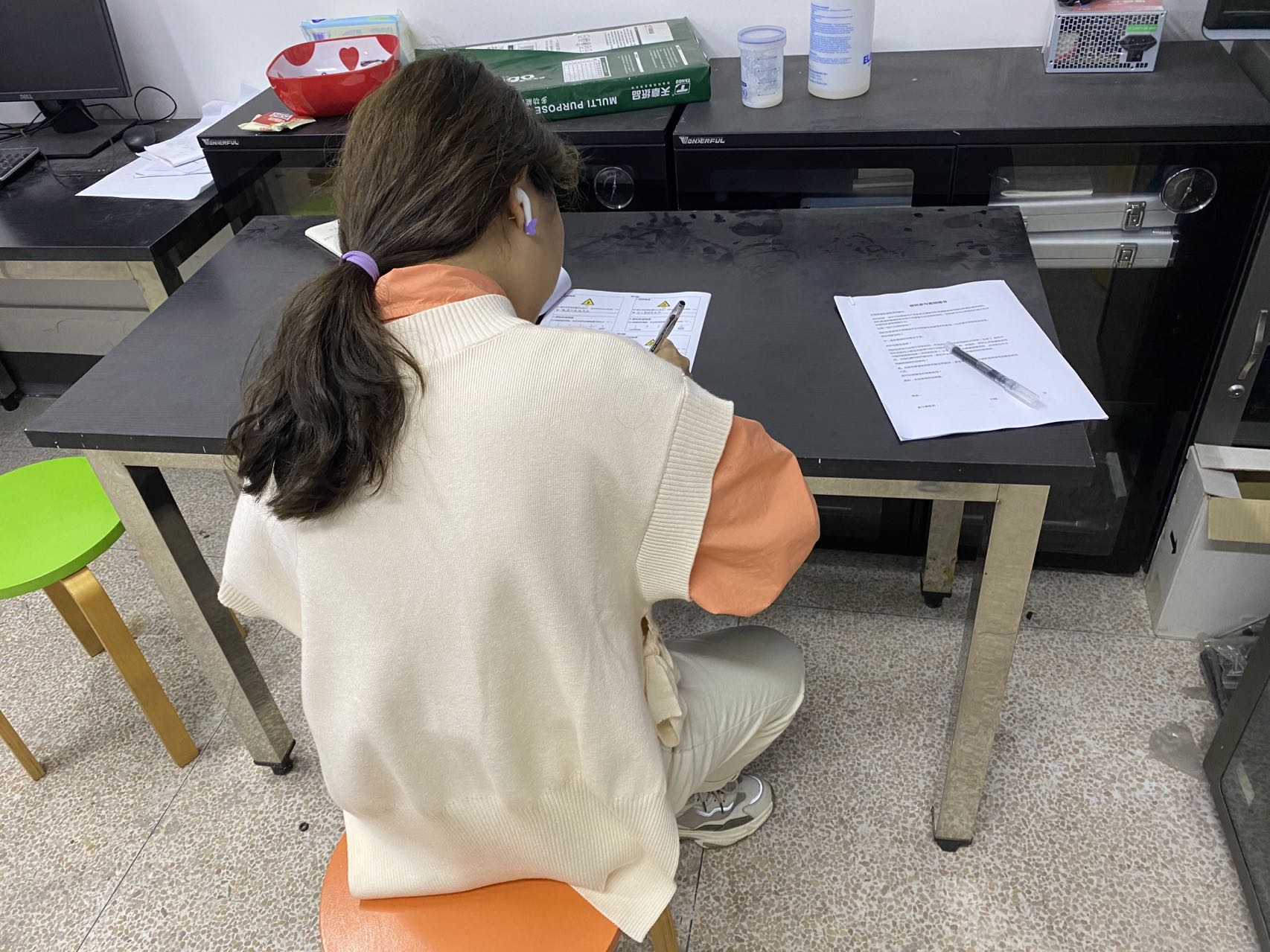

Supplement: Supplementary file 1 [file Data_Sheet_1.ZIP › c0b1915f940d750957a92d28b99b4d8.jpg]

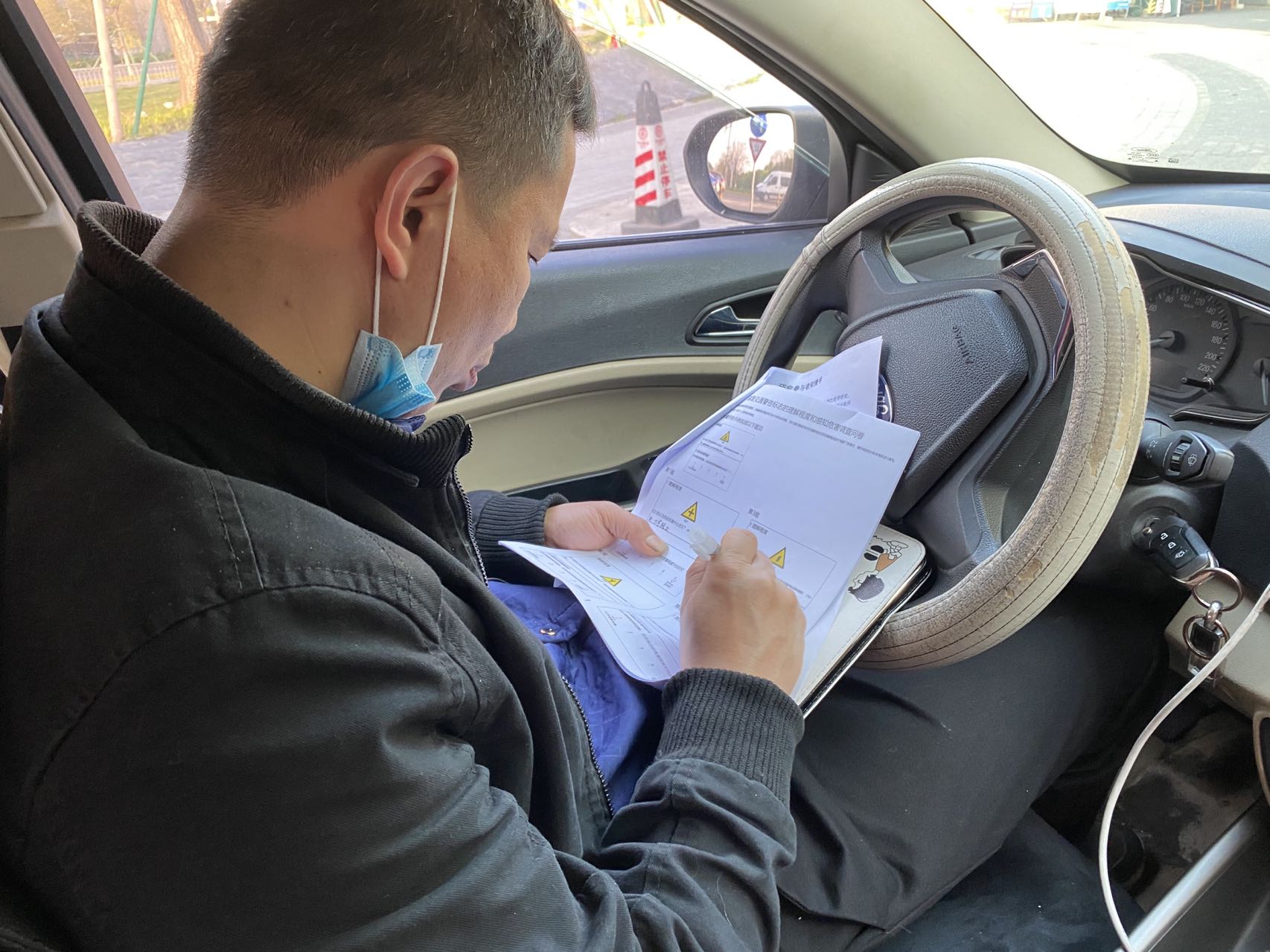

Supplement: Supplementary file 1 [file Data_Sheet_1.ZIP › c836773de1ab9998c56ee6c2d34a1b4.jpg]

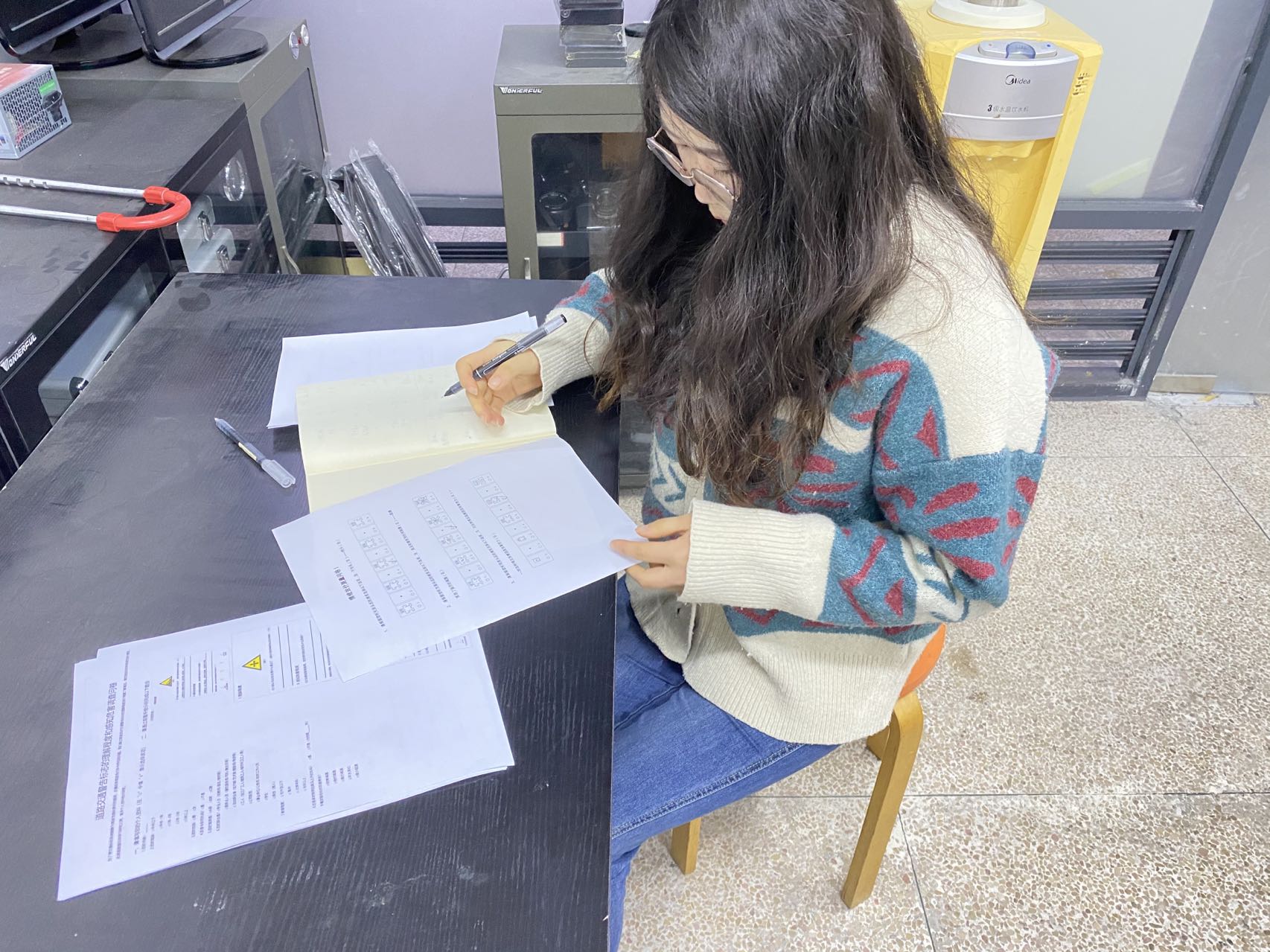

Supplement: Supplementary file 1 [file Data_Sheet_1.ZIP › c98feea191ea4286158f9ca199a6307.jpg]

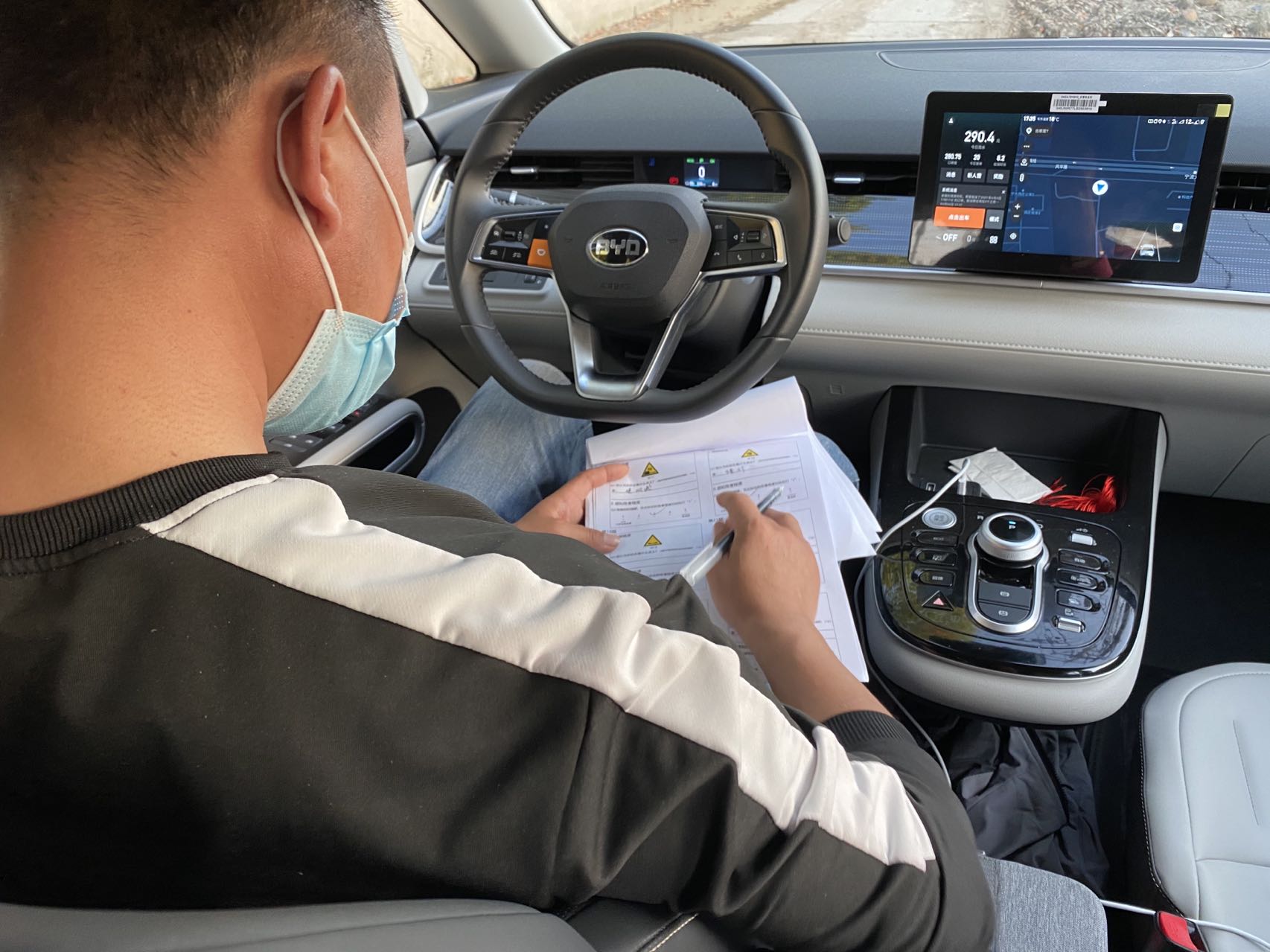

Supplement: Supplementary file 1 [file Data_Sheet_1.ZIP › cdad961ad44f6caa542bb8b3d5b7df3.jpg]

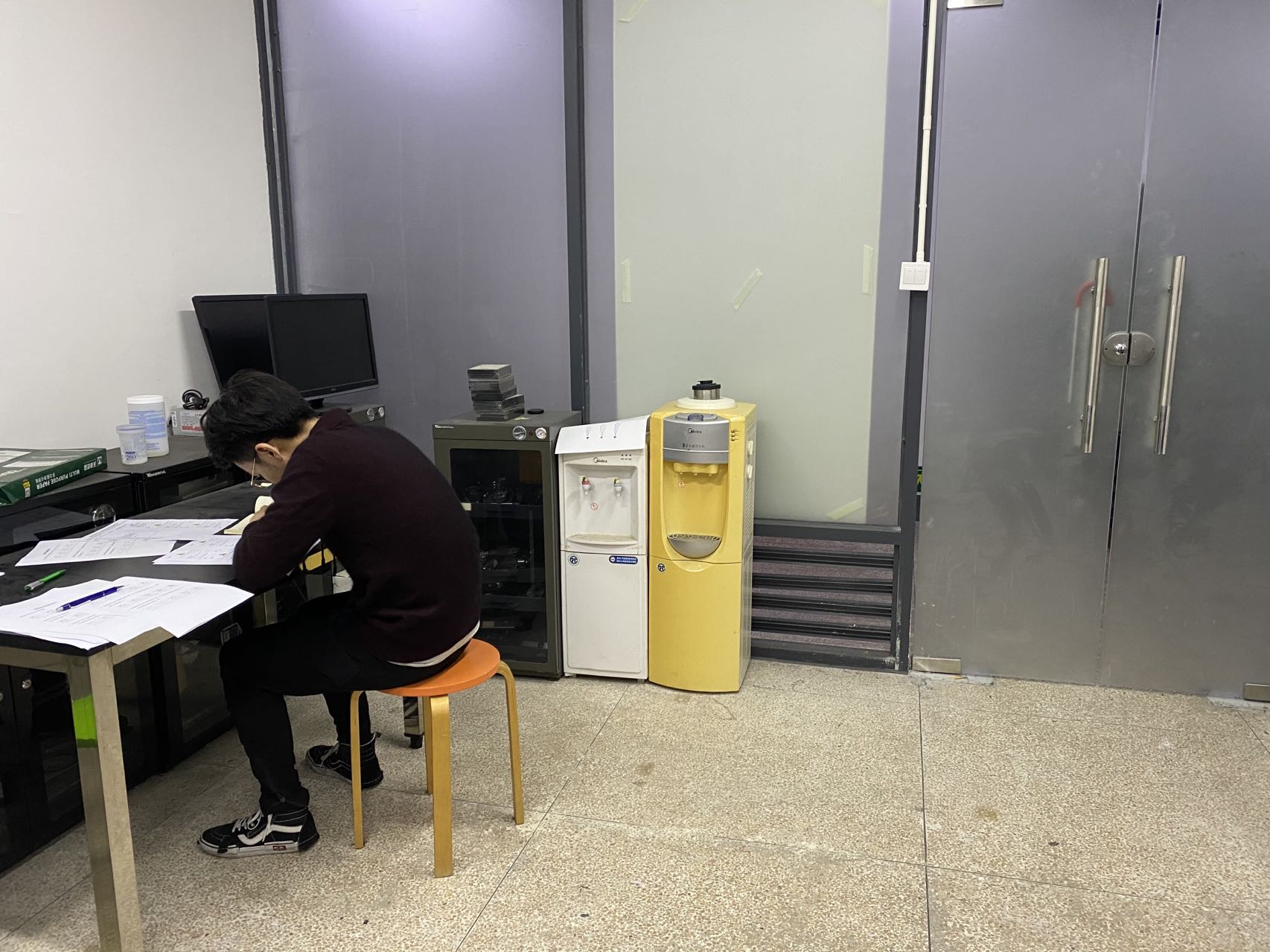

Supplement: Supplementary file 1 [file Data_Sheet_1.ZIP › e130564cbe2845321efcf8fce5175ea.jpg]

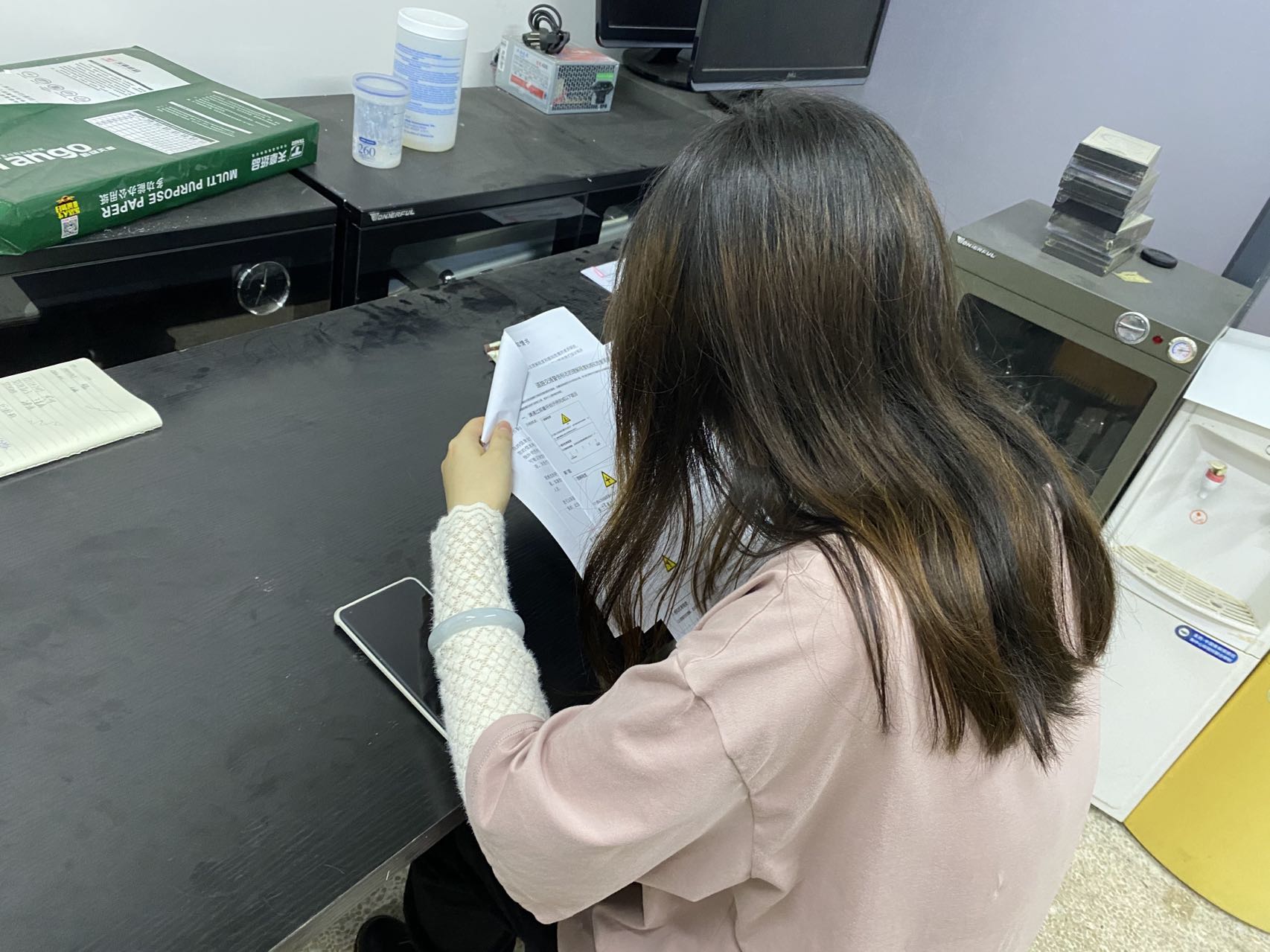

Supplement: Supplementary file 1 [file Data_Sheet_1.ZIP › fc41d8e624767d821e6391427534dad.jpg]
